# Contribution of local climate zones to the thermal environment and energy demand

**DOI:** 10.3389/fpubh.2022.992050

**Published:** 2022-08-09

**Authors:** Ruxin Yang, Jun Yang, Lingen Wang, Xiangming Xiao, Jianhong Xia

**Affiliations:** ^1^Jangho Architecture College, Northeastern University, Shenyang, China; ^2^Human Settlements Research Center, Liaoning Normal University, Dalian, China; ^3^School of Humanities and Law, Northeastern University, Shenyang, China; ^4^Institute of Geographic Sciences and Natural Resources Research, Chinese Academy of Sciences, Beijing, China; ^5^Department of Microbiology and Plant Biology, Center for Spatial Analysis, University of Oklahoma, Norman, OK, United States; ^6^School of Earth and Planetary Sciences (EPS), Curtin University, Perth, WA, Australia

**Keywords:** urban heat island, energy consumption, degree-days, neural network, air temperature inversion

## Abstract

Urban heat islands (UHIs) and their energy consumption are topics of widespread concern. This study used remote sensing images and building and meteorological data as parameters, with reference to Oke's local climate zone (LCZ), to divide urban areas according to the height and density of buildings and land cover types. While analyzing the heat island intensity, the neural network training method was used to obtain temperature data with good temporal as well as spatial resolution. Combining degree-days with the division of LCZs, a more accurate distribution of energy demand can be obtained by different regions. Here, the spatial distribution of buildings in Shenyang, China, and the law of land surface temperature (LST) and energy consumption of different LCZ types, which are related to building height and density, were obtained. The LST and energy consumption were found to be correlated. The highest heat island intensity, i.e., UHILCZ 4, was 8.17°C. The correlation coefficients of LST with building height and density were −0.16 and 0.24, respectively. The correlation between urban cooling energy demand and building height was −0.17, and the correlation between urban cooling energy demand and building density was 0.17. The results indicate that low- and medium-rise buildings consume more cooling energy.

## Introduction

Climate change has become a formidable challenge faced by all countries in the world because of its possible consequences (1). It impacts sea level rise, biodiversity, agricultural production, frequency of extreme weather, human health, and energy demand (2). There is a direct relationship between climate change and energy demand. Climate warming mainly results from the excessive absorption of long-wave radiation from the underlying surface by greenhouse gases (3). Large quantities of fossil fuels are utilized to meet the needs of human production and life processes, resulting in the emission of greenhouse gases (4). Among them, global building energy consumption accounts for ~20 to 40% of total energy consumption and ~33% of greenhouse gas emission (5).

To cope with rising temperatures and manage living standards, residents increase the use of air-conditioning and other refrigeration facilities to reduce indoor temperatures, but these measures in turn intensify the heat island effect. The environmental changes in cities are more complex, owing to many impervious surfaces and human activities (6). High-density buildings change the path and quantity of regional energy absorption and storage, flow and reflection, release, and consumption (7). The response of energy consumption and demand to temperature rise is more complicated. There are differences in the temperature of cities in different regions and scales, at different times and seasons, and even in the distribution characteristics of temperature within cities (8, 9). In general, the growth in expenditure caused by the increase in cooling demand exceeds the savings brought about by the reduction in heating demand, especially at low latitudes. For every 2°C increase in the average land surface temperature (LST), the global net building energy expenditure will increase by 0.1% of the total global economic expenditure (10).

The estimation of energy consumption concentrates on the city scale, and the estimation methods can be divided into two categories: top-down and bottom-up (11). The top-down approach is based on statistics and uses regional urban data to estimate the spatial distribution of energy consumption across cities, based on macroeconomic indicators such as population density, income, energy prices and types, and urban morphology (12). The bottom-up approach is based on each individual building and can be a statistical or physical-based hybrid model (13). Previous research has shown that the net energy impact of the urban environment depends on climate type (14), the urban context density, and functional and structural characteristics of buildings (15).

To improve the accuracy of bottom-up methods, some studies have incorporated local climate into the energy performance simulation of urban buildings. The urban heat island (UHI) effect, which refers to the phenomenon wherein the temperature in cities is higher than that in suburban rural areas (16), is also considered in the estimation of building energy consumption. Palme et al. (17) put forward the method of incorporating the UHI effect into building performance simulation and found that when UHI is incorporated, the energy demand increased by 15–200%. However, simulation methods are often calculation-heavy, and the relationship between urban features and energy consumption is indirect and unclear.

The intensity of heat islands is generally measured by the temperature difference between urban and rural areas. However, there has been no unified statement on how to define urban and rural areas. Over the past century, many scholars have made the division based on population density, building density, and landscape differences, or used gradients to illustrate problems. The duality of urban and rural areas has weakened with the development of cities (18, 19). Stewart and Oke (20) proposed the local climate zone (LCZ) concept to study UHIs, in which the city is divided into different areas according to the height density of buildings and other ground cover. Wong et al. (21) found that the influence of floor area ratio on temperature in Singapore is as high as 2°C, and buildings can save 4.5% on energy consumption by improving urban form. This deconstruction of urban internal structure divides it into organizational units, which can account for the internal form of buildings while segmenting urban areas, and is suitable for studying the influence of urban form on the thermal environment and energy demand.

The scope of energy consumption is highly complex, and all climate changes determined by the urban environment should be considered when performing simulations. For this reason, some coupling techniques between the building energy model and microclimate CFD model have been proposed (22, 23). However, this considers too many measured data (such as climate, wind speed, temperature, building texture, and thermal radiation), which require longer calculation times and higher costs. This is unfavorable for discussing the continuous changes of heating and cooling energy demand in the future. Invidiata and Ghisi (24) also emphasized that meteorological data were not updated in time in the process of estimating indoor air temperature. Obtaining reliable time-sensitive data to evaluate energy consumption is a major challenge in this research.

The scope of energy consumption is highly complex. Cooling energy consumption for the thermal environment under the UHI effect can be calculated from electricity consumption and remote sensing inversion data (25), such as the effective *U*-value method, degree-days, bin method, load frequency table method, equivalent full load hours, weighting factor method, and heat balance method (26). For cities, the degree-days method is simple and suitable for larger areas (27); however, traditional degree-days can only describe a single region at once and cannot analyze the distribution of energy consumption within the entire city. Combined with local climatic zone, the influence of different building forms and land use types on the urban thermal environment and refrigeration energy consumption can be examined in more detail. This macroscopic and easy-to-measure method is more universal and operable.

This new attempt requires more spatial distribution density of meteorological data, and traditional station data cannot meet the requirements. Traditional interpolation analysis using meteorological station data is less accurate. Remote sensing data is appropriate as continuous data. LST data is used frequently for calculations of the surface heat island, whereas for the cooling energy demands of urban dwellers indoors, air temperature is clearly more appropriate. Mesoscale weather Research and Forecasting Model and neighborhood-scale microclimate simulations, for example, are not suitable for urban studies (28). Of the methods that can be applied, regression statistics is the simplest and easiest to build; however, establishing a relevant model depends on the time and place of data acquisition, which may cause large errors. The temperature-vegetation index method is highly dependent on plants; therefore, it is not suitable for cities with a large impermeable surface coverage. The surface energy balance method has good portability and universality, but many physical parameters needed by the model cannot be obtained directly by remote sensing, and the data cannot be updated in real time. Such models yield sufficiently accurate estimates, provided that all the required parameters are involved in the calculation. The parameters is not always possible, and thus, the data-driven model was selected. Neural network machine learning relies on large amounts of sample data, which can experience difficult modeling problems starting as a black box and express the nonlinear relationship between LST and air temperature (29, 30).

This study used remote sensing images, building data, and meteorological data as parameters and referred to Oke's LCZ method to divide urban areas according to the height and density of buildings and land cover types. The research attempted to apply neural networks for retrieving air temperature to calculate cooling energy demand, combining the number of degree days with the division of local climatic zones. The aims of the study were to improve energy demand structure of space and provide a relevant reference for urban planning under the prerequisite of ensuring the comfort of urban residents.

## Materials and methods

### Study area

**Figure 1:**
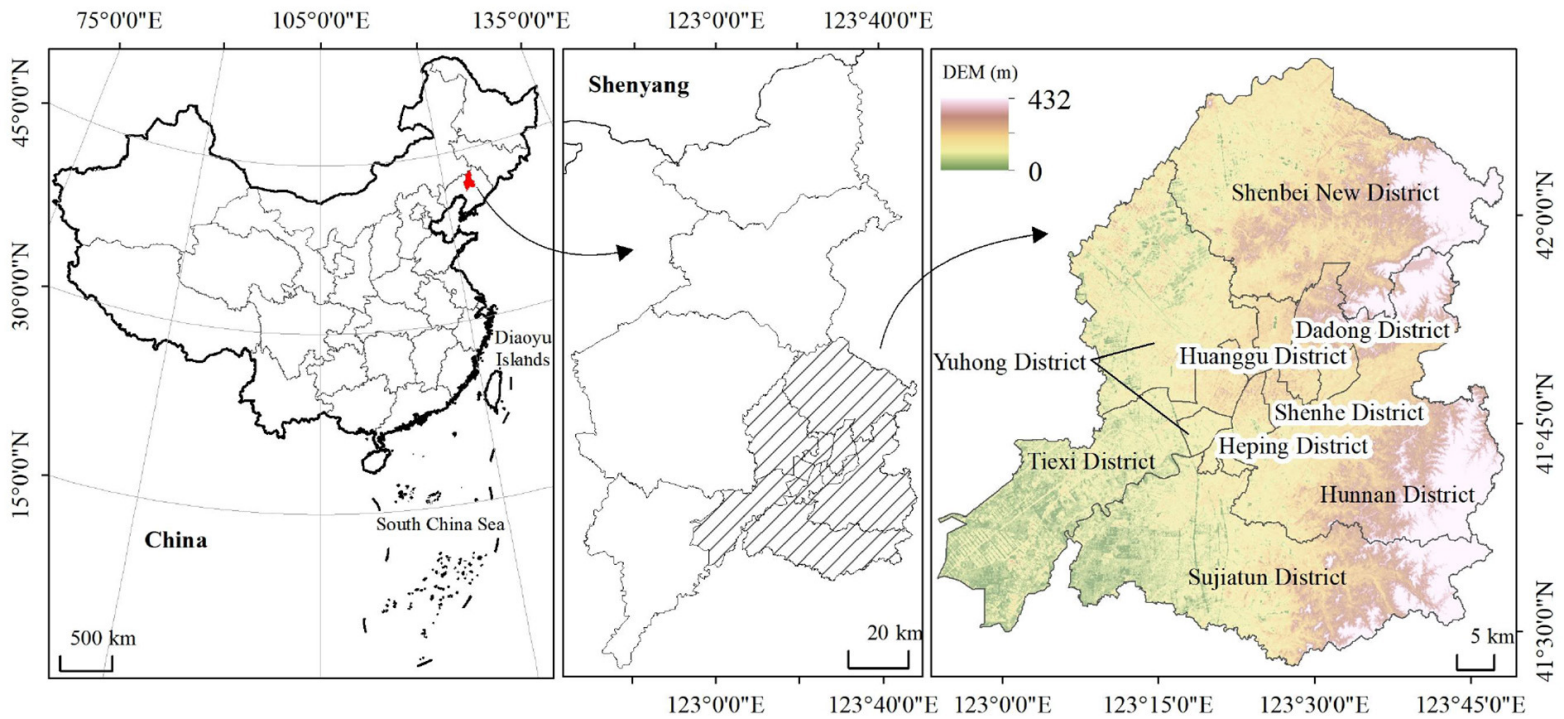
The location of the study area.

Shenyang City (Figure 1) is located in Northeast China, central Liaoning (122°24′59″-123°48′30 E; 43°2′25″-41°11′53″N). The terrain gradually changes from hills in the northeast to plains in the southwest. The region has a temperate, semi-humid continental climate. Rainfall is concentrated in the summer months, but the area is sunnier than South China, making it conducive to the acquisition of remote sensing images. It is the political, economic, and cultural center of the Northeast region, as well as an important transportation hub. The building types are diverse and comprised of varying building height densities. Human activities have affected this area, leading to local climate change. In recent years, the annual maximum temperature in Shenyang exceeded 38°C in the summer.

### Data sources and processing

The data used mainly included MODIS remote sensing, temperature, building outline, land cover, and administrative division. Table 1 contains detailed information on the remote sensing platforms and related data processing. In terms of time selection, according to the “Uniform Standard for Civil Building Design” GB 50352-2019, building climate zoning uses the average temperature in July as the main reference for summer. In traditional Chinese solar terms, dog days, the days of highest temperature generally start in mid-July. Considering the availability of data, the study period ranged from mid-July to early August. The technical flowchart is shown in Supplementary Figure 1.

**Table 1:** Data sources and descriptions.

**Type**	**Description**	**Time**	**Resolution**	**Data sources**	**Process**
Remote sensing data	MODIS MOD11A2 (land surface temperature products)	2018.07.12–20180.8.04	1 km	https://search.earthdata.nasa.gov	Calculate the average daily maximum land surface temperature within the study time range.
	MOIDS MOD13A3 (vegetation Index Products)		1 km		
	MODIS MCD43C3 (surface albedo products)		0.05 deg		Obtain the black and white sky albedo in the shortwave band (0.3~5.0μm), also noon solar altitude angle.
	Landsat 8		OLI 30 m TIRS 100 m	USGS https://glovis.usgs.gov	
Meteorological data	Daily maximum temperature			http://www.resdc.cn/	The average daily maximum temperature corresponding to the study tie.
Elevation	ASTER GDEM V2	2018	30 m	China Academy of Sciences http://www.gscloud.cn	Data splicing and clipping
Building outline	Building outline and floor number	2018	–	Baidu Map	Calculate the height of buildings, and find the height and density of the building within a 30 m grid.
Land cover	Land use cover type	2018	30 m	http://www.resdc.cn/	–
Administrative boundary	–	2020	–	–	–

### Local climate zone

The urban form and nature of the surface are one of the main reasons for the difference between the urban LST and air temperature. In this study, we referred the definitions of Stewart and Oke (20) to divide the city into LCZ A–G according to the types of natural cover (Detailed classification diagrams are in the Supplementary material). Vertically, the height of the building class was divided from low-rise to super high-rise buildings at consistent intervals of three floors. Horizontally, the building densities were divided into dense and open building groups with a boundary of 40%. Accordingly, LCZ 1–10 were obtained (31). Finally, 17 categories were obtained, as shown in Supplementary Table 1. The height (BH) and density (BD) of the buildings were calculated on a 30 × 30 m grid using Eqs. (1) and (2).


(1) $$\begin{array}{r} BH=\frac{\overset{n}{\underset{i=1}{\sum }}H_{i}}{n} \end{array}$$

(2) $$\begin{array}{r} BD=\frac{\overset{n}{\underset{i=1}{\sum }}S_{building}}{S_{grid}} \end{array}$$

Where n represents the number of single buildings in a grid, *H*_*i*_ represents the number of floors of the *i*-th building in a grid, *S*_*grid*_ represents the area of a grid, and *S*_*building*_ represents the base area of all buildings in the grid.

### Heat island effect intensity

The LST was retrieved using Tan Zhihao's single-window algorithm, and Landsat was selected for heat island intensity analysis (32) (see the calculation section of the Supplementary materials). The intensity of the heat island effect is usually measured by the temperature difference between urban and rural areas. Stewart and Oke combined this temperature difference with the local climate zoning of the city and defined the formula of UHI intensity as the difference between a certain type of LCZ (X) and LCZ D (low vegetation, such as grass) (33). The intensity of the heat island effect is shown by Eq. (3).


(3) $$\begin{array}{r} UHI_{LCZX}=T_{LCZX}-T_{LCZD} \end{array}$$

Where *UHI*_*LCZX*_ and *T*_*LCZX*_represent the heat island effect intensity and LST of a certain type of LCZ X, respectively, and *T*_*LCZD*_ represents the LST of the LCZ D type.

### Cooling energy demand

Using the method of combining cooling degree-days (CDD) (34) with LCZ, we calculated the change in building energy demand for different types of LCZ at different summer temperatures. The basic formula is shown in Eq. (5). According to previous experience, 23–24°C is the most comfortable ambient temperature for human life (35), and the difference between the interior design temperature of a building and the heat balance temperature of the building is usually 3–7°C (36). Finally, the base temperature of the cooling day was determined to be 26°C.


(4) $$\begin{array}{r} CDD26={\sum_{days}\left(t-t_{ref}\right)}^{+} \end{array}$$

Where *CDD26* represents the number of cooling degree-days with 26°C as the base temperature, *t* is the average temperature of approximately a month, and *t*_*ref*_ is the base temperature.

Weather station data are generally used when calculating the number of degree-days. However, the meteorological station data were discrete point data, and were not detailed enough for research on urban interior building patterns. Therefore, we used remote sensing images in this study to retrieve air temperature as the basic temperature data calculated by CCD to evaluate the energy demand of buildings in a typical month.

Considering the accuracy and difficulty of calculation, the four factors, namely remote sensing albedo, normalized difference vegetation index (NDVI), elevation, and LST, were selected (37–39). The meteorological station data in the study area were used as the verification data, and the neural network regression model (40, 41) was used to train a model for inversion of air temperature. After several attempts, the training algorithm was finally settled on Levenberg-Marquardt, a toolbox already written in Matlab. For MODIS MCD43C3 products, the daily surface albedo α of the short-wave band can be calculated using Eqs. (5) and (6) (42–44). Finally, the formula for calculating the building energy demand under a certain type of LCZ is shown in Eq. (7).


(5) $$\begin{array}{r} \alpha =r\alpha_{w}+\left(1-r\right)\alpha_{B} \end{array}$$

(6) $$\begin{array}{r} r=0.122+0.5\exp\left(-4.8\cos\theta \right) \end{array}$$

(7) $$\begin{array}{r} CDD26={\left(t_{LCZX}-26\right)}^{+} \end{array}$$

In the formulas, α_*W*_ represents the albedo of the white sky, α_*B*_represents the albedo of the black sky, *r* represents the ratio of the sky scattered radiation to the downward solar radiation, and θ represents the solar zenith angle of the study area at noon. The values required in Eqs. (5) and (6) are given in MODIS MCD43C3. *t*_*LCZX*_ is the average air temperature within the study time range of X under the LCZ system.

## Results

### Spatial pattern distribution of local climate zones

**Figure 2:**
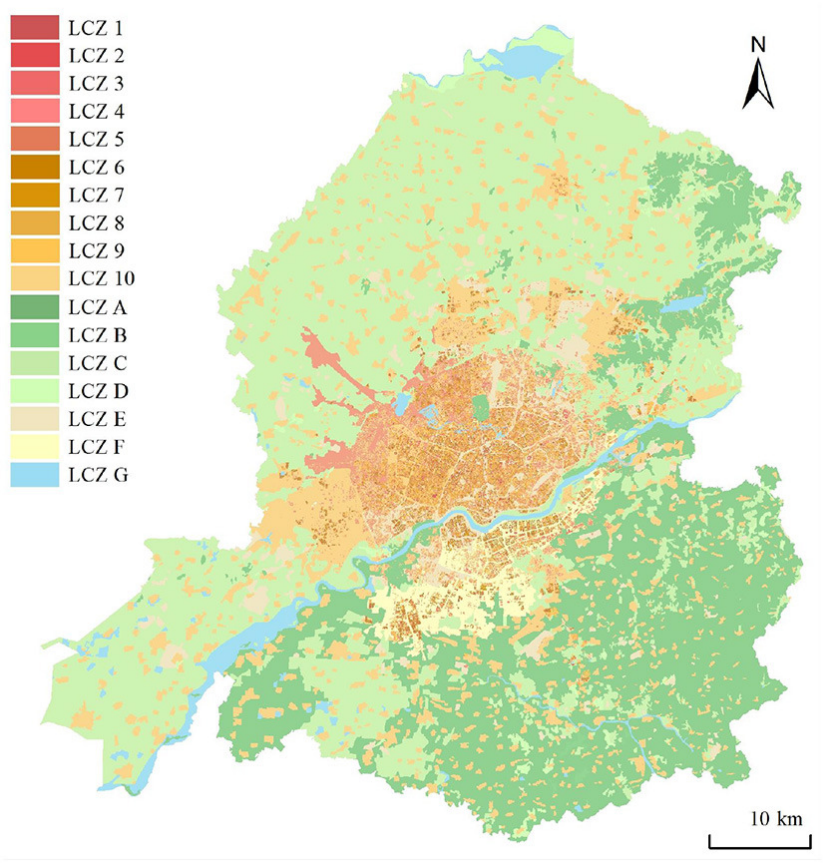
LCZ distribution in study area. LCZ, Local climate zone.

The main urban area of Shenyang is dominated by low-rise buildings, which are widely and evenly distributed, followed by the middle-rise and mid-high-rise buildings, which are concentrated in the center of the main urban area. The number of buildings below 40% density is evenly distributed, rising steeply up to 40%, reaching a peak, and then slowly falling. In terms of spatial distribution, urban buildings were high-density and low-density, with low-density buildings dominating, but with no specific area of concentration. The LCZ distributions are shown in Figure 2.

LCZ 10 accounted for 11.69% and featured the largest proportion of building coverage due to the large number of villages outside the built-up area; LCZ 9 ranked second, accounting for 1.66%, and contained mostly residential land. LCZ 2 accounted for the smallest proportion (0.94%) and was concentrated in the city center, around the factory, near the high-speed railway station, and on the south bank of the Hunhe River. The proportion of LCZ 7 was slightly higher than that of LCZ 2. Natural cover was dominated by cultivated land in the northwest and lush forest land in the southeast, accounting for 39.21 and 25.99%, respectively; sparse forest land accounted for the lowest coverage (0.30%). The city center of Shenyang is surrounded by arable land and woodland, but the center itself has less green space and is crowded by buildings. The surrounding development requires strengthening.

### Urban thermal environment space

**Figure 3:**
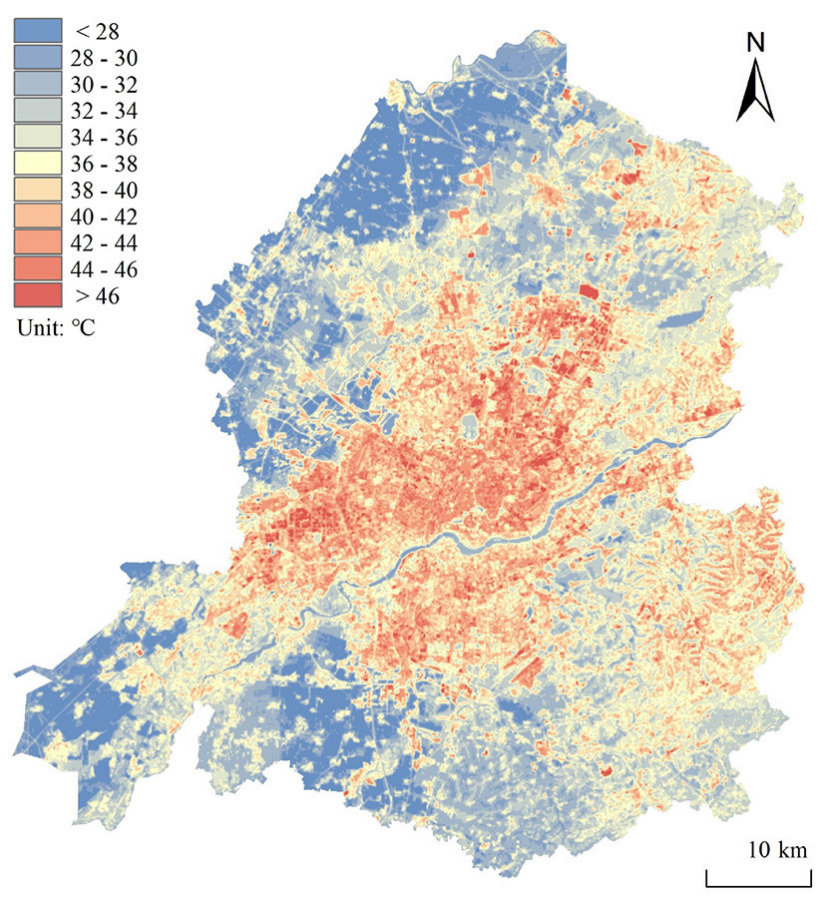
Land surface temperature distribution.

**Figure 4:**
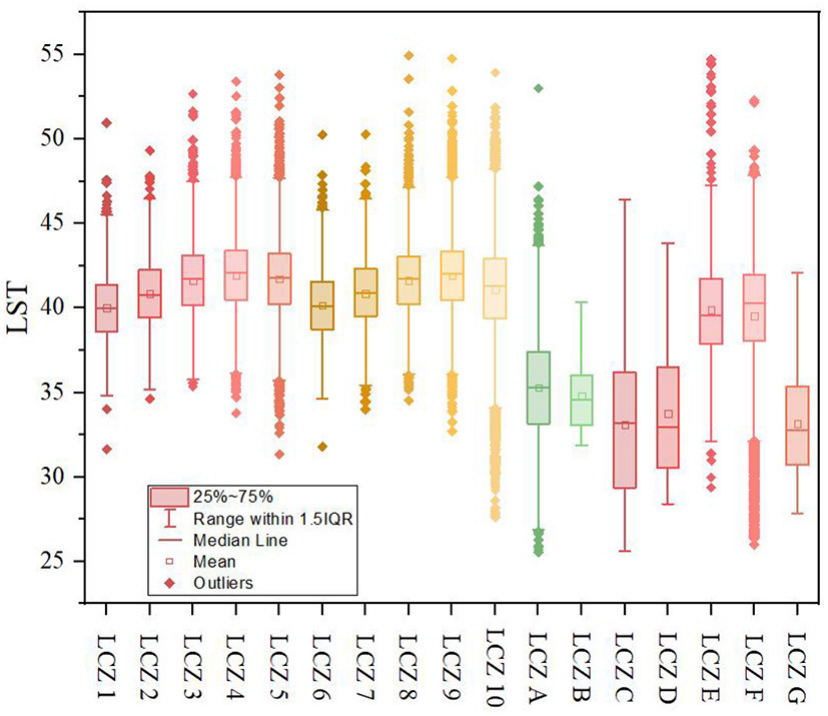
Distribution of LST in LCZ (LST unit:°C). LST, land surface temperature. LCZ, local climate zone. The results are sampled and counted according to the calculation results associated with the LCZ grids.

The LST (Figure 3) of the building coverage was generally higher than that of the natural coverage. The average temperature of various LCZ overall displayed the following distribution law: LCZ 4 > LCZ 9 > LCZ 5 > LCZ 8 > LCZ 3 > LCZ 10 > LCZ 7 > LCZ 2 > LCZ 6 > LCZ 1 > LCZ E > LCZ F > LCZ A > LCZ B > LCZ D > LCZ G > LCZ C (Figure 4).

Among them, the temperature of LCZ D is 33.77°C. The highest heat island strength, UHI_LCZ__4_, was 8.17°C, and the lowest, UHI_LCZC_, was −0.68. The lowest heat island strength of the building coverage UHI_LCZ1_ was 6.23°C. The LST changes of LCZ 1 were the most stable, with a standard deviation of 1.96, while LCZ G showed the most dramatic changes, with a standard deviation of 4.11, and the standard deviations of the other types were concentrated in the range of 2.8 ± 0.96. LST was negatively correlated with building height, with a Pearson correlation coefficient of −0.16, reaching significance at the 0.01 level, and positively correlated with building density, with a correlation coefficient of 0.24, reaching significance at the 0.01 level. These results can be explained by the fact that although higher buildings accumulate artificial materials and create more light reflections in three dimensions, they also cast larger shadows on the ground, which reduce the LST.

### Urban cooling energy demand

In neural network estimation of air temperature, 70% of the data were randomly selected as the training sample, 15% as the verification sample, and 15% as the test sample to invert the air temperature. The model mean square error was RMSE = 0.32, R = 0.92, and R^2^ = 0.84 (Supplementary Figure 4). According to these data, the overall range of air temperature was included in the distribution interval of the LST, and the value was between 28 and 33°C. The various types of air temperature change ranges were relatively stable and exhibited low volatility.

**Figure 5:**
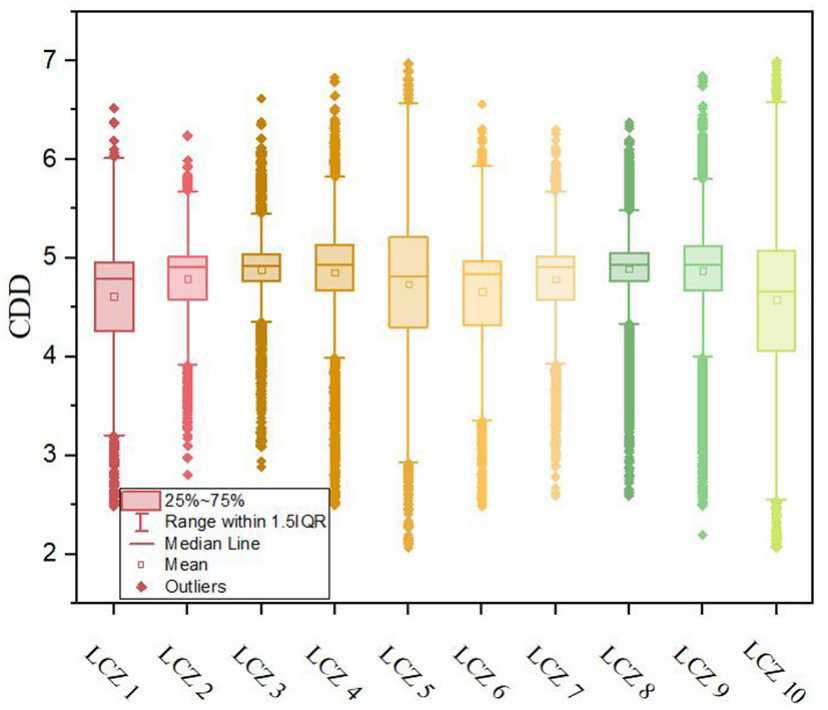
Distribution of CDD in LCZ (CDD unit:°C d). CDD, cooling degree-days. The results are sampled and counted according to the calculation results associated with the grid of the different LCZ plots.

The average energy consumption of the building covering generally presented the distribution law of LCZ 8 > LCZ 3 > LCZ 9 > LCZ 4 > LCZ 2 > LCZ 7 > LCZ 5 > LCZ 6 > LCZ 1 > LCZ 10 (Figure 5). Among them, the maximum value of CDD was 7.00°C · d, the lowest value was 2.06°C · d, and the standard deviation of the different building coverage types varied between 0.232 and 0.76°C · d. The Pearson correlation between urban cooling energy demand and building height reached −0.17, and the correlation with building density was 0.17, both of which passed the 0.01 level two-tailed significance test. Compared with the distribution of cooling energy demand, LST is more affected by building height and density.

## Discussion

### Impact of local climatic zones on cities

**Figure 6:**
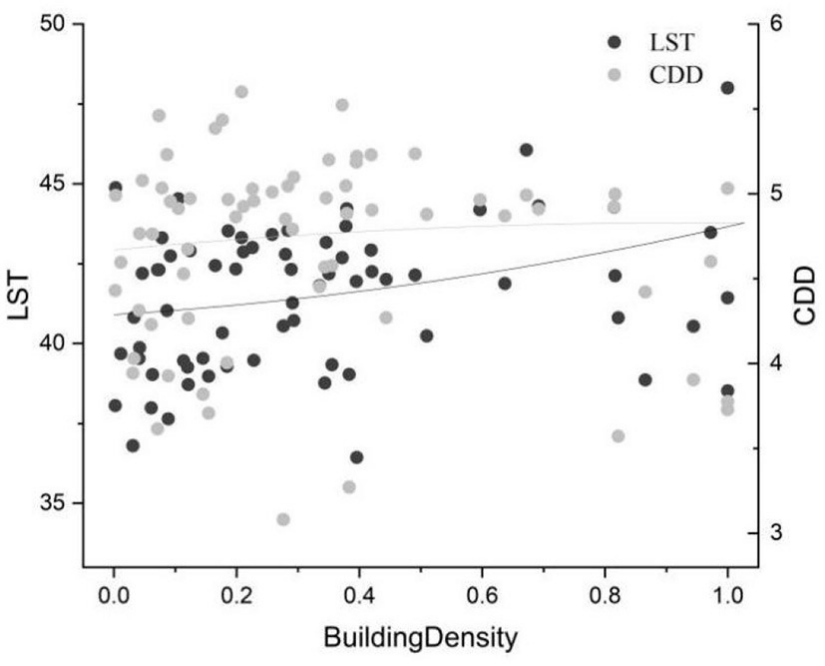
Correlation between LST (CDD) and building density (LST unit:°C).

**Figure 7:**
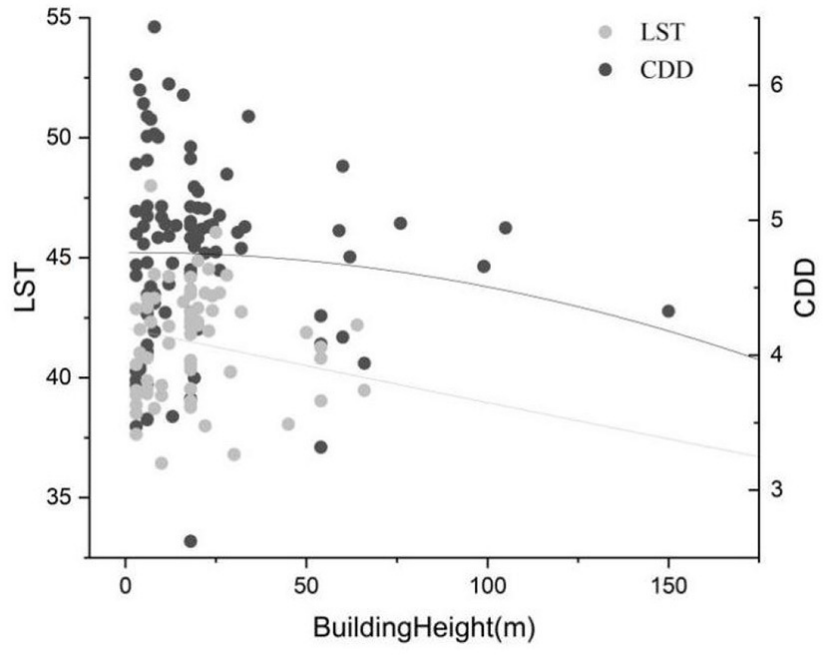
Correlation between LST (CDD) and building height (CDD unit:°C d).

The distribution of air temperature is more concentrated than that of LST, and the temperature is lower; however, the distribution difference between different local climatic zones is not so obvious. LST and cooling energy demand are positively correlated with building density. Compared with cooling energy demand, LST is more affected by building density (Figure 6); however, there is a weak negative correlation between LST and building height (Figure 7). The negative correlation may be explained by the addition of super high-rise buildings (43–45). Although the taller buildings accumulate man-made materials and produce more light reflection in three dimensions, they also cast larger shadows on the ground, thus lowering the LST. The correlation with building height is not as high as that with building density because of these complex relationships.

**Figure 8:**
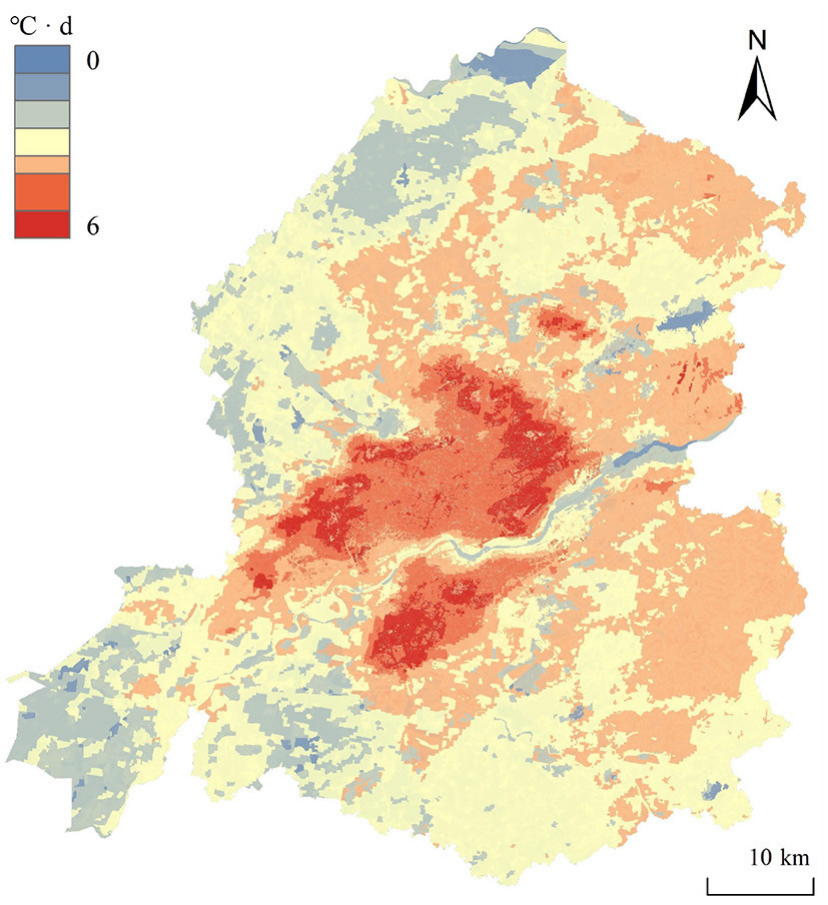
CDD of study area. CDD, cooling degree-days.

Figure 8 shows that the CDD value gradually increases from the outskirts of the city to the center, but the range greater than 5.0°C · d surrounds the range of 4.5–5.0°C · d, showing a concave distribution. This phenomenon was attributed to the effects of the Hun River, which flows east-to west and appears in the south-central part of the study area, and Youth Street that runs north to south and is located in the middle of the city. The summer winds blow north through the Hunhe River and pass through Qingnian Street, forming a ventilation corridor that somewhat controls the regional temperature and reduces the potential cooling energy demand (46). Based on the dark red area in Figure 8, for general planning we recommend that coverage by green spaces (47, 48) or water bodies is increased to reduce energy consumption, and LCZ8, LCZ3, and LCZ9 should be focused on and reduced.

The difference of air temperature is not as large as that of land surface temperature, which also shows the importance of the material selection of the underlying surface from the side. It is recommended to increase vegetation and water area, sprinkle water on the road, and appropriately reduce the density of urban buildings to reduce the temperature.

### Limitations

The Landsat image for heat island intensity analysis has relatively high resolution. Although Landsat's temporal resolution cannot meet the needs of daily data in the study time range, the spatial distribution of LST in sunny weather is still representative.

As a calculation method of cooling energy demand, the degree-days are simple and effective in characterizing urban energy; however, this method does not fully consider the effect of individual building differences (49, 50). For the study of overall characteristics, these parameters are simplified in the modeling process, but for urban planning, these factors are closely integrated with energy conservation.

The accuracy of the model used in this study was mainly limited by the remote sensing data and the number of meteorological stations in the study area. Because the calculation of energy consumption requires a high temporal resolution of remotely sensed images, MODIS products were finally selected from the available data (51, 52). The air temperature retrieved by combining various factors can show the characteristics of distribution in space, but the improvement of the initial data quality can greatly improve the accuracy of the results. Due to the limited availability of data, this study selected provincial capital cities with richer building data for analysis; however, there are still missing data in some areas. Future studies should focus on other thermal environment characteristic indicators, simultaneously consider the energy consumption requirements of cooling and heating, and use long time series and high-resolution data.

## Conclusions

This study used remote sensing images, building data, meteorological data, and other basic data, referring to Oke's LCZ, to divide urban areas according to the height and density of buildings and land cover types, and explored the distribution characteristics of energy consumption that may be generated by urban residents' refrigeration demand in different regional thermal environments. The following conclusions were obtained.

The main urban area of Shenyang is dominated by low-rise buildings, which are widely and evenly distributed, and mid-high-rise buildings, which are crowded in the center.

The distribution of air temperature is more concentrated than that of LST, and the temperature is lower; thus, the difference in the distribution among the various local climatic zones is not evident.

The LST of the building covering is generally higher than that of the natural covering. The average temperature overall displays the distribution law as LCZ 4 > LCZ 9 > LCZ 5 > LCZ 8 > LCZ 3 > LCZ 10 > LCZ 7 > LCZ 2 > LCZ 6 > LCZ 1> LCZ E > LCZ F > LCZ A > LCZ B > LCZ D > LCZ G > LCZ C. Among them, the temperature of LCZ D is 33.77°C. The highest heat island intensity of UHI_LCZ4_ was 8.17°C. The LST is negatively correlated with building height, with a correlation coefficient of −0.16, reaching significance at a level of 0.01; and a positive correlation with building density, with a correlation coefficient of 0.24, reaching significance at the 0.01 level.

The average energy consumption of the building covering generally presents the distribution law of LCZ 8 > LCZ 3 > LCZ 9 > LCZ 4 > LCZ 2 > LCZ 7 > LCZ 5 > LCZ 6 > LCZ 1 > LCZ 10. Among them, the maximum value of CDD was 6.997°C · d, and the lowest value was 2.06°C · d. The correlation between urban cooling energy demand and building height reached −0.17, and the correlation between urban cooling energy demand and building density was 0.17, with both correlation coefficients shown to be statistically significant through a two-tailed significance test (p < 0.01).

## Data availability statement

The original contributions presented in the study are included in the article/Supplementary material, further inquiries can be directed to the corresponding author/s.

## Author contributions

RY wrote the main manuscript text. JY directed and revised the manuscript and contributed to all aspects of this work. LW, XX, and JX conducted the experiment and analyzed the data. All authors reviewed the manuscript.

## Funding

This research study was supported by the National Natural Science Foundation of China (Grant Nos. 41771178 and 42030409), the Fundamental Research Funds for the Central Universities (Grant No. N2111003), Basic Scientific Research Project (Key Project) of the Education Department of Liaoning Province (Grant No. LJKZ0964), and Innovative Talents Support Program of Liaoning Province (Grant No. LR2017017).

## Conflict of interest

The authors declare that the research was conducted in the absence of any commercial or financial relationships that could be construed as a potential conflict of interest.

## Publisher's note

All claims expressed in this article are solely those of the authors and do not necessarily represent those of their affiliated organizations, or those of the publisher, the editors and the reviewers. Any product that may be evaluated in this article, or claim that may be made by its manufacturer, is not guaranteed or endorsed by the publisher.
