# Supplementary material for: Contribution of local climate zones to the thermal environment and energy demand
Source: Front Public Health. 2022 Aug 9;10:992050. doi: 10.3389/fpubh.2022.992050 (PMC9395604; doi:10.3389/fpubh.2022.992050)
Supplement: Supplementary file 1 [file Data_Sheet_1.docx]

**
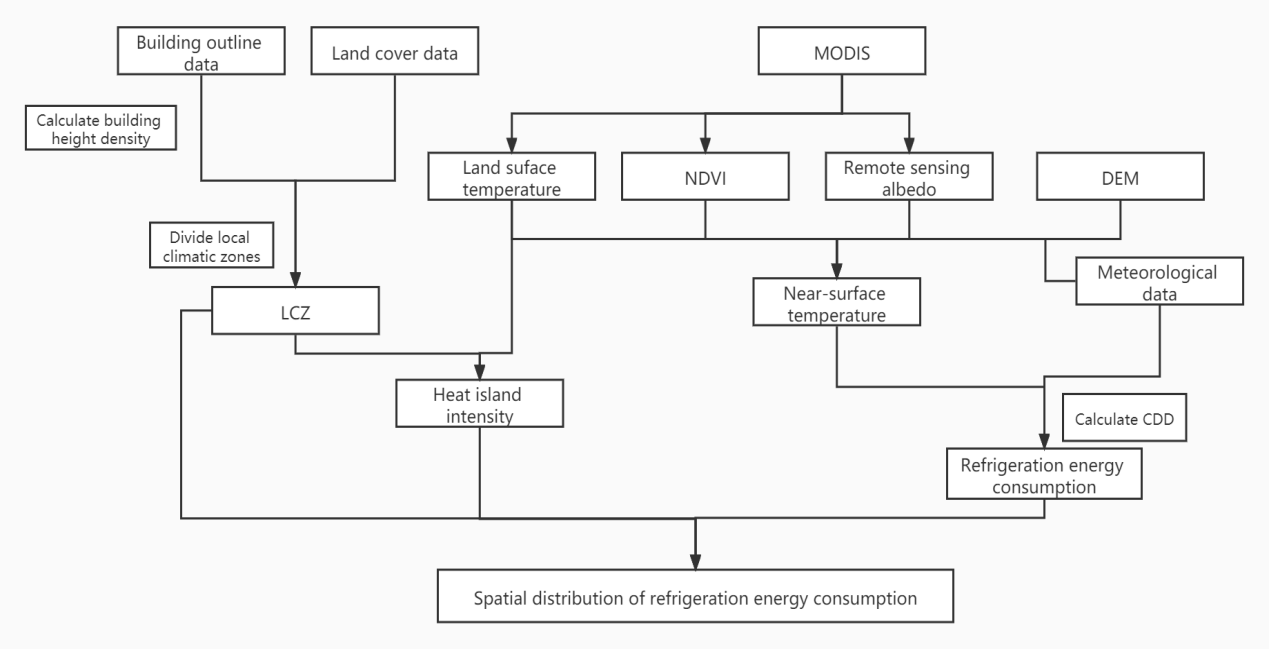
**

**Supplementary Figure 1.** The technical flow chart.


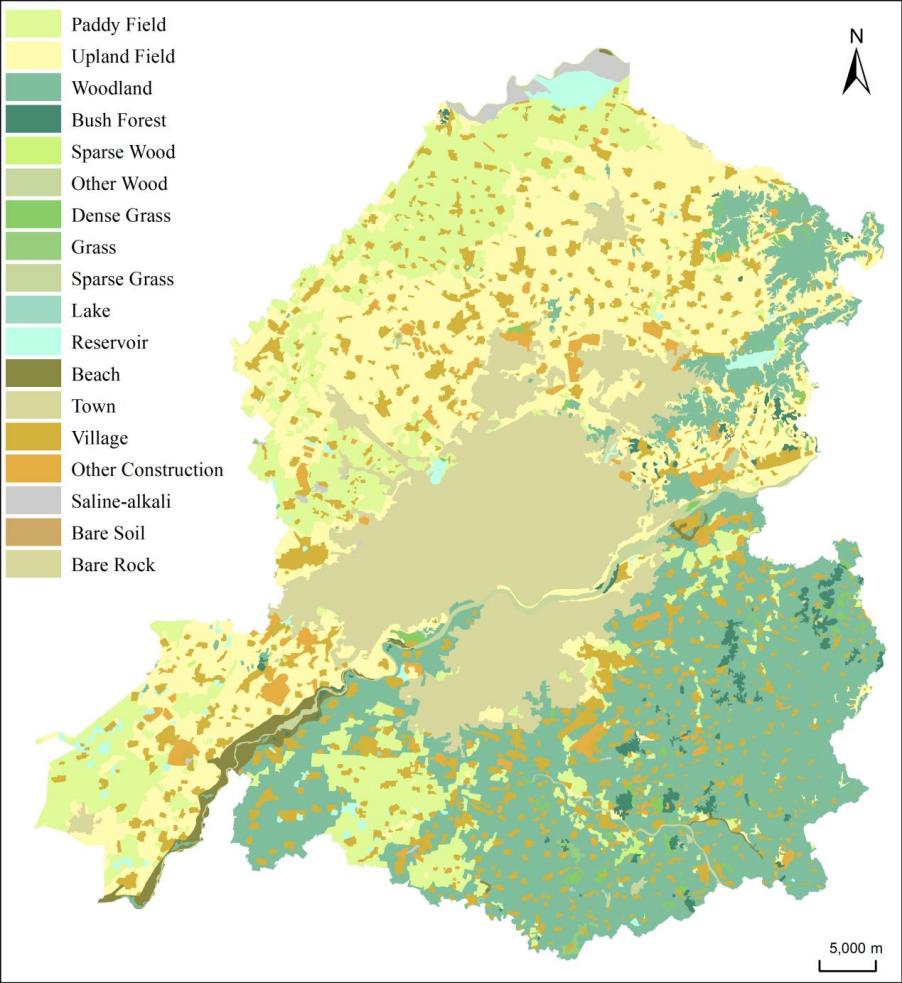


**Supplementary Figure 2.** Land cover in study area.

**Supplementary Table 1.** Data sources and descriptions.

| Series | Description | Instances | Series | Description | Instances |
| --- | --- | --- | --- | --- | --- |
| LCZ 1 | Compact super high-rise  (Above 12 floors) | 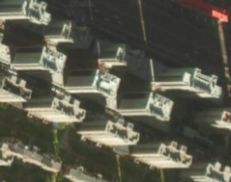 | LCZ 10 | Open low-rise  (1-3 floors) | 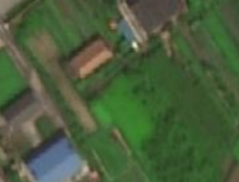 |
| LCZ 2 | Compact high-rise  (10- 12 floors) | 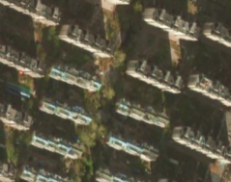 | LCZ A | Dense trees  (Natural forests and plantations with canopy closure> 30%) | 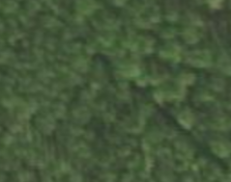 |
| LCZ 3 | Compact middle-high-rise  (7- 9 floors) | 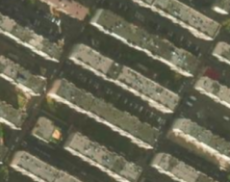 | LCZ B | Scattered trees  (Deciduous forest, evergreen forest, etc. with a canopy density of 10-30%) | 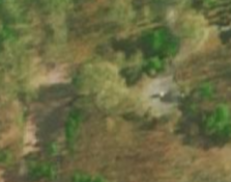 |
| LCZ 4 | Compact mid-rise  (4- 6 floors) | 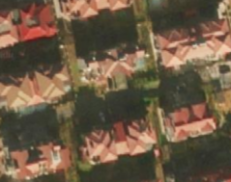 | LCZ C | Bush(canopy closure> 40% ,low woodlands and shrubs with a height of less than 2m) | 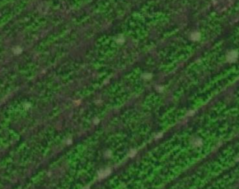 |
| LCZ 5 | Compact low-rise  (1-3 floors) | 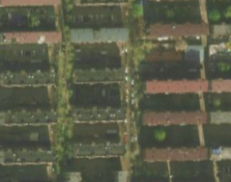 | LCZ D | Grass  (Low herbs, etc.) | 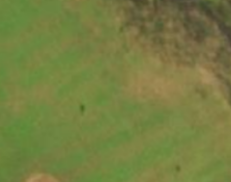 |
| LCZ 6 | Open super high-rise  (Above 12 floors) | 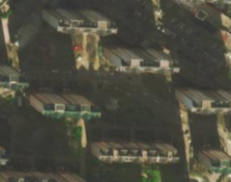 | LCZ E | Bare rock and paved | 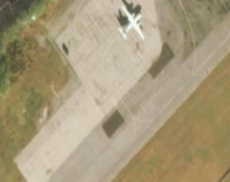 |
| LCZ 7 | Open high-rise  (10- 12 floors) | 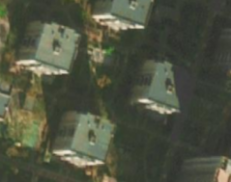 | LCZ F | Bare soil and sand | 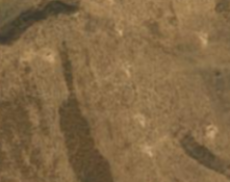 |
| LCZ 8 | Open middle-high-rise  (7- 9 floors) | 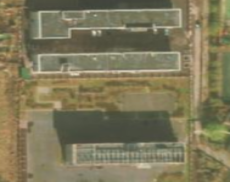 | LCZ G | Water (natural waters and artificial water conservancy facilities) | 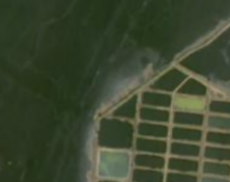 |
| LCZ 9 | Open mid-rise  (4- 6 floors) | 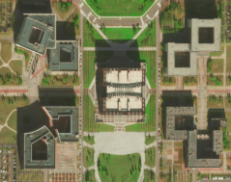 | - | - | - |

**Surface Temperature Retrieval**

Tan Zhihao's single-window algorithm was used to inversion the average LST corresponding to the time, which produced highly accurate results with very few parameters, and it incorporated surface and atmospheric effects directly into the algorithm. The basic formula of LST(*T_s_*, unit: °C) is shown in Eq. (S1)-(S3).

$T_{s}=\frac{a(1-C-D)+\left[ (b-1)(1-C-D)+1 \right]T_{b}-DT_{a}}{C}+273.15$ (S1)

$C=\varepsilon\tau$ (S2)

$D=(1-\varepsilon)\left[ 1-(1-\varepsilon)\tau\right]$(S3)

where *a* and *b* are constants, and the value are - 67.355351 and 0.458606, respectively. Tb is the brightness temperature (K); *T_a_* is the effective mean atmospheric temperature (K); *C* and *D* are

intermediate variables; $\varepsilon$ is the atmospheric transmittance; and $\tau$represents the transmittance of atmosphere in thermal infrared band. The $\tau$ value was shown in Figure S3, calculated according to the atmospheric parameters on NASA’s official website.


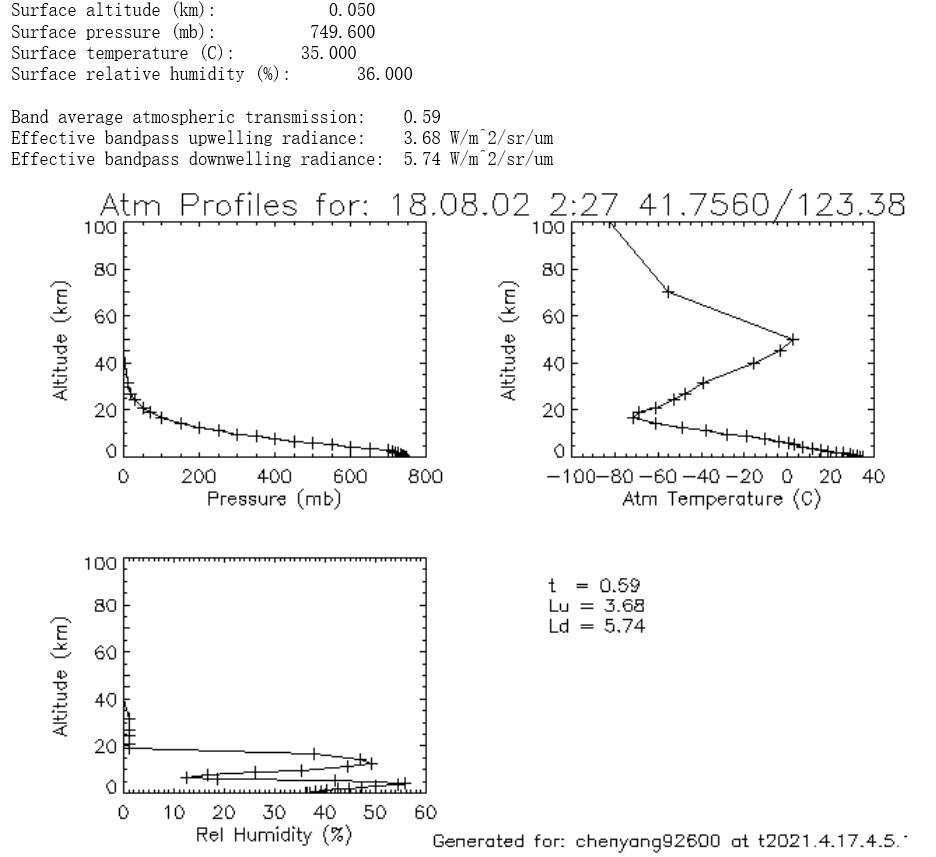


**Supplementary Figure 3.** the transmittance calculation of atmosphere in thermal infrared band.


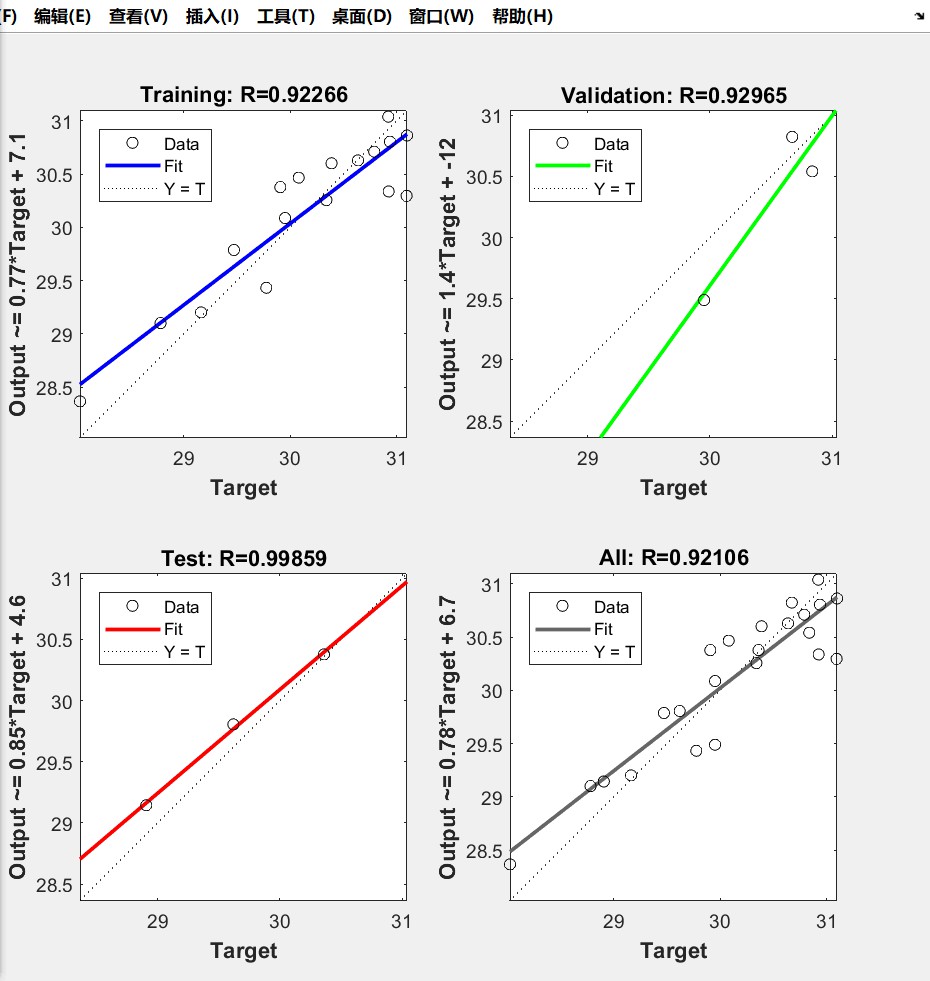


**Supplementary Figure 4.** neural net fitting result.
